# Monthly Botanical Report

**Published:** 1810-11

**Authors:** 


### MONTHLY BOTANICAL REPORT.

We have seen, with much pleasure, an essay upon the natural order of the *Scitamineæ*, by Dr. Roxburgh, printed at Calcutta. The doctor has for the most part adopted the genera of Mr. Roscoe, but from his long residence in the East Indies, and his situation in the Company's botanic garden, he has had a much greater opportunity of examining numerous species of these plants in a living state, than could possibly fall to the lot of any botanist resident in Europe. He has of course availed himself of these opportunities in making some corrections and many additions to Mr. Roscoe's essay. For want of the plates, however, with which this work is to be illustrated, but which are not yet arrived, we cannot at present make any critical examination of it.

In the volume of the Hortus Kewensis, recently published, we observe, in referring to the Botanical Magazine, the author has been careful to distinguish, by his mode of quoting that work, between the original work, as published by Mr. Curtis himself, and its continuation by his successors. In the former case the work is always quoted *Curtis Magaz.* in the latter, *Botan. Magaz.* [In the explanation of *Botan. Magaz.* in the list of books quoted, we observe a trifling error, *James* instead of *John Sims*; which ought, however, to be corrected, as both names occur in the list of London physicians. J. Bellenden Ker is very properly added with a parenthesis, as one of the authors of this work; for though his name does not occur in the title-page, very nearly half of the articles are from his pen, and are marked with the letter G. Gawler being his name at the time he commenced writing in the magazine.] When any new name or specific character is adopted into the Hortus Kewensis from the Botanical Magazine, the names of Ker or Sims are added.

We

We observe little new in the class Diandria, except the addition of new species, the specific characters of which are for the most part taken from Wildenow, Vahl, &c. without alteration. We were rather surprized to observe that the number of species of Veronica is just the same (37) as in the prior edition. In Justicia the species are increased from 9 to 28; in Zizephora from 3 to 6; Salvia from 42 to 59; Piper from 5 to 17; Valeriana from 12 to 19.

In the class Triandria much interesting and new matter occurs. This class contains the principal part of the natural order of *Ensatæ*, a family that has been much studied by Mr. Bellenden Ker, and explained at considerable length in the Annals of Botany; and the species more fully described in the Botanical Magazine.

Mr. Dryander has adopted the whole of Mr. Ker's genera, but the characters of the latter being prolix in the extreme, not formed at all in the concise manner of Linnæus, and rather descriptions than definitions, he has, in a masterly manner, framed new generic and specific characters upon the Linnæan plan; certainly with sufficient brevity, yet, as far as we have had an opportunity of examining, adequate to the task of distinguishing the known species one from another. The discovery of additional species may indeed hereafter render these definitions inefficient; for it is impossible to frame perfect characters till all the species are known, the discovery of a new species possessing the same characters as used in the definition of one already known, yet different from it, will of course make it necessary to alter the specific phrase of the latter so as to exclude the new found species also.

In this class many of the genera have received a great addition of species since the prior edition. Crocus, which in that had only two varieties, in this contains nine species, of which six flower in the spring, and three in the autumn. Ixia, being divided into several new genera, viz. Trichonema, Glissorhiza, Hesperantha, Sparaxis, Anomatheca, Tritonia, Babiana, Lapeyronsia, Pardanthus, and Ixia Proper, is reduced in the latter from 18 to 13 species, but increased on the whole to 52. Gladiolus is increased from 10 to 23 species, though some of the former are removed to other genera. There are ten species of Watsonia, a genus originally framed by Miller, and established by Ker, most of which were before referred to Gladiolus or Ixia. Moræa and Iris have undergone a new arrangement by Mr. Ker, which is generally followed here, and twenty species are added to the two genera. In Marica Mr. Dryander has deviated from Mr. Ker, the species enumerated by the former are only *Northiana*, *martinicensis*, and *paludosa*; *plicata*, *striata*, and *californica* of the latter, are probably reserved to be subjoined to Sisyrinchium, a genus according to Ker not to be distinguished from Marica, but arranged in the Linnæan system under *Gynandria triandria*. It will be evident from the above short account that much new matter occurs in *Triandria Monogynia*, and the whole appears to have been very carefully got up by the author, and will, we doubt not, be highly acceptable to botanists in general, abroad as well as at home.

In the same class are the natural orders of *Cyperoideæ* and *Gramineæ*. Amongst the former we observe that Vahl's new genus of Rhyncospora is adopted, which includes Schœnus *albus* and *fuscus*, of Linnæus. Many new species are added to most of the genera in both orders.

In the class *Tetrandria* occurs such part of the natural order of Proteaceæ as have hermaphrodite flowers; the Linnæan system unfortunately demands the separation of this family; those genera which have monoecious or dioecious flowers are of course postponed to the classes *Monœcia* and *Diœcia*. In this order Mr. Dryander has entirely followed Mr. Brown's essay on the *Proteaceæ* in the 10th volume of the Transactions of the Linnæan Society, with scarcely any alteration, further than the term *corolla* is adopted for the *calyx* of Mr. Brown and Jussieu, and here and there a superfluous word is omitted. Undoubtedly our author could not have followed a better guide than Mr. Brown, whose knowledge of the *proteaceæ* is greater than that of any man; yet we cannot but feel a wish that Mr. Dryander had undertaken to revise the specific charaoters, and given them more of the Linnæan terseness and precision. We acknowledge that the task would be difficult, for in a perfectly natural order, where the species of a genus are numerous, the difference is frequently marked rather by a number of points of slight deviation, than by any striking feature; nevertheless, though not easy to be accomplished, we do think that had he undertaken it, all obstacles would have been surmounted by his abilities.

To show how great a number of new plants of this family have of late years been introduced into this country, we need only observe that the former edition of the work under review contained only twenty-four species, whereas the new one contains one hundred and fourteen, divided into seventeen genera; though a considerable proportion of the family are deferred to a future part.

With the genera of Mr. Brown more liberty has been taken; all of them have undergone a revision. To shew the mode in which this is done we shall transcribe the generic character Protea as given by both authors. By Brown. *Calix* bipartibilis inæqualis, labii latioris laminis staminiferis cohærentibus. *Stylus* subulatus. *Stigma* angustius, cylindraceum. *Nux* undique barbata, stylo persistenti caudata. *Receptaculum*, commune paleis abbreviatis persistentibus. *Involucrum* imbricatum persistens. By Dryander. Petala quatuor, quorum tria superne cohærencia. *Antheræ* apicibus concavis corollæ immersæ. *Nux* supera, undique barbata, stylo persistente coronata.

In the remainder of the class *Tetrandria* we observe that the specific characters are in general the same as in Willdenow, except in a few species not found in that author; three or four in the genus Pethos, and the whole of Struthiola, for which new specific phrases have been adopted.

In Petandria Monogynia, (not yet finished, as the volume ends with Strychnos), there is a number of this species enumerated, which have been introduced since the former edition; in most of these the specific characters of Willdenow are followed. The only deviations we have observed are as follow:

Cynoglossum *sylvaticum* is adopted from Dr. Smith; Echium *grandiflorum* from Ventenat; E. *parviflorum* from Roth; Symphytum *asperrimum*, and Onosma *taurica* from Sims. Echium *fastuosum*, native of the Canary Islands, is new. In Androsace *villosa*, Dr. Sims is followed, and Menyanthes *exaltata* and Lysimachia *quadriflora* are adopted from the the same author. Primula *nivalis* of Pallas, and *longifolia* of Curtis, are both inserted, though according to Dr. Sims they are the same species. Epacris, Andersonia, Styphelia and Leucopogon, are New Holland genera, and the characters of Mr. Brown, in his Prodromus, are used exclusively. Plumbago *tristis* is a new species, as is Phlox *prostrata*. P. *pyramidalis* is admitted from Smith, and *stolonifera* from Sims; as are Convolvulus *erubescens* and *bryonio folius* of the same. C. *pannifolius* of Salisbury, C. *suffruticosus* is new. Ipomopris of Michaux and Smith is taken up. As are Cobœa of Cavanilles, Campanula *versicolor* of Andrews and Smith. C. *collina* of Sims, Phyteuma *campanuloides*, Lobelia *gracilis* and L. *bicolor* of the same, L. *alata* of Labillardiere. In Goodenia, Scævola, Euthales and Samolus, the characters of Brown are adopted. It is remarkable that Samolus *Valerandi*, a native of Europe, is found also in New Holland. Rondeletia *lævigata* and *hirta* are new, as is Mussæada *pubescens*. Oxyanthus of Decandolle and Pinckneya of Michaux are adopted ; as are Nicotiana *undulata* and Verbascum *ovalifolium* of Sims. In V. *Lychnitis*, V. *pulverulenta*, V. *virgatum*. V. *Blattaria* Dr. Smith's characters are used. As are those of Cavanilles for Solanum *betaceum ;* and of Poiret for S. *Lyracantha*. Physalis *pubescens* is inserted with the synonym of Feuillée, which Dr. Sims says belongs to his P. *edulis*, a species not admitted. Are both the last mentioned species then to be considered as the same ? Strychnos *agmina claudit*.

When we wrote the above Report, Mr. Dryander, altho' incommoded by local complaint, not considered in the least dangerous, was in good general health, and in the full possession of his great mental powers ; but alas, already he is no more ; and we, in common with all lovers of natural science, have to deplore a loss, that will be severely felt in the scientific world, as a public calamity ; and to those who, from a personal acquaintance with him, had a knowledge of his worth, will cause the most poignant regret. In the situation he held, as librarian to Sir Joseph Banks, the loss will, we fear, be in great measure irreparable. His head was stored with knowledge, beyond that of almost any man, and not confined to his more immediate pursuits, but in the wide-extended range of science in general ; even in political and in personal history it was most extensive. This knowledge he was very ready to impart, where he thought it would be useful, being very communicative to enquirers of this stamp, though repulsive to impertinent curiosity, and possessing little of that suavity of manners for which his predecessor was so eminent. He neither fawned upon nor flattered any one ; but ever spoke truth without blushing. It grieves us to think that this stupendous store-house of knowledge, this living cyclopædia, is gone ; and, like the baseless fabric of a vision, has scarcely left a wreck behind.

———precipe lugubres
Cantus, Melpomene !

